# Enhanced Identification of Postoperative Infections among Inpatients

**DOI:** 10.3201/eid1011.040572

**Published:** 2004-11

**Authors:** Deborah S. Yokoe, Gary A. Noskin, Susan M. Cunningham, Gianna Zuccotti, Theresa Plaskett, Victoria J. Fraser, Margaret A. Olsen, Jerome I. Tokars, Steven Solomon, Trish M. Perl, Sara E. Cosgrove, Richard S. Tilson, Maurice Greenbaum, David C. Hooper, Kenneth E. Sands, John Tully, Loreen A. Herwaldt, Daniel J. Diekema, Edward S. Wong, Michael Climo, Richard Platt

**Affiliations:** * Brigham and Women's Hospital, Boston, Massachusetts, USA;; † Northwestern University, Chicago, Illinois, USA;; ‡ Memorial Sloan-Kettering Cancer Center, New York, New York, USA;; § Washington University School of Medicine, St. Louis, Missouri, USA;; ¶ Centers for Disease Control and Prevention, Atlanta, Georgia, USA;; # Johns Hopkins Medical Institutions, Baltimore, Maryland, USA;; ** North Shore Medical Center-Salem Hospital, Salem, Massachusetts, USA;; †† Massachusetts General Hospital, Boston, Massachusetts, USA;; ‡‡ Beth Israel Deaconess Medical Center, Boston, Massachusetts, USA;; §§ Mount Auburn Medical Center, Cambridge, Massachusetts, USA;; ¶¶ University of Iowa Carver College of Medicine, Iowa City, Iowa, USA;; ## McGuire Veterans Affairs Medical Center, Richmond, Virginia, USA;; *** Harvard Pilgrim Health Care, Boston, Massachusetts, USA

**Keywords:** Surgical wound infection, postoperative complications, surveillance, drug utilization, cross-infection, nosocomial infection, coronary artery bypass, cesarean section, mastectomy, research

## Abstract

We evaluated antimicrobial exposure, discharge diagnoses, or both to identify surgical site infections (SSI). This retrospective cohort study in 13 hospitals involved weighted, random samples of records from 8,739 coronary artery bypass graft (CABG) procedures, 7,399 cesarean deliveries, and 6,175 breast procedures. We compared routine surveillance to detection through inpatient antimicrobial exposure (>9 days for CABG, >2 days for cesareans, and >6 days for breast procedures), discharge diagnoses, or both. Together, all methods identified SSI after 7.4% of CABG, 5.0% of cesareans, and 2.0% of breast procedures. Antimicrobial exposure had the highest sensitivity, 88%–91%, compared with routine surveillance, 38%–64%. Diagnosis codes improved sensitivity of detection of antimicrobial exposure after cesareans. Record review confirmed SSI after 31% to 38% of procedures that met antimicrobial surveillance criteria. Sufficient antimicrobial exposure days, together with diagnosis codes for cesareans, identified more postoperative SSI than routine surveillance methods. This screening method was efficient, readily standardized, and suitable for most hospitals.

## Abstract 2

Monitoring antimicrobial exposure and diagnosis codes for certain procedures identifies more postoperative infections than routine surveillance methods.

Preventing healthcare-associated infections, including surgical site infections (SSI), is an integral part of the national patient safety agenda developed in response to the Institute of Medicine's report, To Err is Human: Building a Safer Health System (*1*). Although nearly all hospitals monitor their SSI rates, no generally accepted active surveillance method is both reproducible and widely used. Fewer than 10% of hospitals participate in the Centers for Disease Control and Prevention's (CDC) National Nosocomial Infection Surveillance System (NNIS). Many hospitals use surveillance systems based more or less closely on NNIS; most of these systems are labor-intensive and their sensitivity is typically unknown.

We therefore studied the ability of exposure to antimicrobial drugs and coded discharge diagnoses to identify SSI after three common procedures: coronary artery bypass graft (CABG) procedures, cesarean delivery, and breast procedures. We chose these measures because prior work suggested their usefulness (*2**,**3*), nearly all hospitals collect this information as part of routine patient care, and many hospitals store this information electronically.

## Methods

We conducted this study in two phases in 13 hospitals affiliated with the seven CDC epicenters. Phase 1 involved seven teaching and community hospitals in eastern Massachusetts. Phase 2 involved hospitals in seven states. The study design was similar for each of the procedures, although the antimicrobial intervals required to trigger record review were procedure-specific and based on earlier work (*2**,**3*). We describe the methods in detail for CABG procedures and then provide additional information for cesarean delivery and breast procedures. The number of hospitals assessing the three procedures differed because of different practice patterns.

### CABG Procedures

#### Phase 1–Eastern Massachusetts Epicenter

We studied consecutive CABG procedures performed from April 1, 1998, through January 31, 1999, in four hospitals. Qualifying International Classification of Diseases 9th Revision Clinical Modification (ICD-9-CM) procedure codes are listed in Table A1, Table A2, Table A3, Table A4, Table A5, Table A6, Table A7, Table A8, Table A9, and Table A10. Infection control professionals performed prospective inpatient SSI surveillance, typically once or twice per week, using the NNIS definitions (*4*). The surgical sites usually were not observed directly by the infection control professionals. We included SSI detected during the initial hospital admission for the CABG procedure or any readmission to the same hospital that occurred within 60 days of the operative procedure. Weeks or months later, the hospitals' information systems and medical records were used to obtain information about antimicrobial drug exposure and discharge diagnoses.

#### Discharge Diagnosis Codes Screening and Antimicrobial Exposure Screening

We identified patients with ICD-9-CM discharge diagnosis codes that suggest SSI (see Tables A1-A10) from their initial hospitalization or readmission to the same hospital within 60 days of the operative procedure. We identified patients for whom >9 days elapsed from the first to the last postoperative dates on which they received parenteral or oral antimicrobial drugs (i.e., an antimicrobial interval of >9 days), based on the results of prior work (*2*). The first postoperative day was ignored because antimicrobial prophylaxis was typically given at that time. Patients did not need to receive antimicrobial agents on each day or the same agent throughout the interval. For instance, a patient could meet the 9-day criterion by receiving cefazolin on the second postoperative day, no antimicrobial drug on postoperative days 3 through 10, then receive an oral quinolone on postoperative day 11. This definition simplified data collection and is nearly as efficient as a measure that required the same antimicrobial drug to be administered continuously during the interval (*2*). We also identified patients who received any oral or parenteral antimicrobial drug during a readmission to the same hospital within 60 days of the operative procedure. Antiviral drugs and antifungal agents were excluded.

#### Identification through Antimicrobial Exposure or ICD-9-CM Code and Interreviewer Reliability

Infection control professionals reviewed the medical records of all patients who met either the antimicrobial drug exposure or ICD-9-CM diagnosis code criteria but who had not been classified as infected during prospective NNIS-based surveillance. The infection control professionals used NNIS criteria to classify the patients' infection status. An NNIS-trained infection control professional who was not affiliated with any of the participating hospitals also reviewed the medical records of all patients who were classified as not having an SSI by routine surveillance but who met diagnosis code, antimicrobial drug exposure screening criteria, or both.

### Analysis and Statistical Methods

Incidence rates of SSI, the sensitivity and the positive predictive value of original prospective surveillance, antimicrobial drug exposure screening, diagnosis code–screening, and combinations of these were determined against the standard criterion created by combining the SSI detected prospectively during routine surveillance and the SSI identified retrospectively when medical records were reassessed. We defined the positive predictive value of routine prospective surveillance to be 100%. Logistic regression was used to evaluate whether diagnosis codes and antimicrobial drug exposure were performed homogenously across the participating hospitals. The κ statistic was used to assess agreement in SSI classification between hospital-based infection control professionals and the external reviewer (SAS software, v. 8, SAS Institute, Cary, NC)

#### Phase 2–All Epicenters

One hospital participated from each of the six epicenters that performed CABG procedures. One hospital had also participated in phase 1. The study population included consecutive CABG procedures performed from July 1, 1999, through June 30, 2001, in the six hospitals.

The methods described for phase 1 were also used for phase 2, except that research personnel retrospectively reviewed the medical records of all patients who had been classified by routine surveillance as having SSI. In addition, research personnel also reviewed the medical records of a random sample of 200 patients who underwent CABG procedures at each hospital to identify patients with ICD-9-CM diagnosis codes suggestive of SSI, to extract antimicrobial drug exposure data, and to retrospectively reassess each patient for the presence of an SSI.

The total number of SSI was estimated by multiplying the SSI rate identified by medical record review in the random sample of patients not known to have SSI based on routine surveillance by the number of patients in the entire group who were not known to have SSI. This estimate was added to the number of SSI identified through routine surveillance to estimate an adjusted SSI rate for each hospital. The sensitivity and positive predictive value of original prospective surveillance, screening by antimicrobial drug intervals, screening by diagnosis codes, and combinations of these were estimated by using the known sampling fractions to extrapolate to the source population. Binomial confidence intervals (CI) were calculated for SSI rates on the basis of the random sample and extrapolated to the source population (Stata software, v. 8.2, Stata Corporation, College Station, TX). Logistic regression was used to evaluate whether screening for SSI by diagnosis code and antimicrobial drug exposure was performed homogenously across the participating hospitals (SAS software, version 8, SAS Institute, Cary, NC).

### Cesarean Deliveries

#### Phase 1–Eastern Massachusetts Epicenter

We studied consecutive cesarean deliveries performed from April 1, 1998, through January 31, 1999, in five hospitals. Qualifying procedure codes are listed in Table A1, Table A2, Table A3, Table A4, Table A5, Table A6, Table A7, Table A8, Table A9, and Table A10. Each participating hospital performed routine inpatient SSI surveillance. We identified patients assigned an ICD-9-CM diagnosis code that suggested SSI (Table A1, Table A2, Table A3, Table A4, Table A5, Table A6, Table A7, Table A8, Table A9, and Table A10), who met antimicrobial drug exposure criteria during the initial hospital admission or any readmission to the same hospital within 60 days of the operative procedure, or both. We used an antimicrobial drug interval of >2 days for cesarean deliveries. Medical record review and analysis were performed as described.

#### Phase 2–All Epicenters

One hospital from each of four epicenters evaluated cesarean deliveries occurring from July 1, 1999, through June 30, 2001. Two hospitals used the methods described. In two other hospitals that had not performed routine prospective inpatient surveillance for SSI, research personnel retrospectively reviewed the medical records of all patients who met either ICD-9-CM diagnosis code or antimicrobial exposure criteria, plus records from a random sample of 200 other patients.

The total number of SSI was estimated as described. For the hospitals without routine surveillance, the total number of SSI was estimated by multiplying the SSI rate in the random sample by the number of patients in the entire group who did not meet diagnosis code or antimicrobial exposure criteria. This estimate was added to the number of SSI identified through medical record review of patients who met diagnosis code or antimicrobial drug exposure criteria to calculate an estimated adjusted SSI rate for each hospital.

### Breast Procedures

#### Phase 1–Eastern Massachusetts Epicenter

We studied consecutive breast procedures performed from April 1, 1998, through January 31, 1999, in seven hospitals (see procedure codes in Table A1, Table A2, Table A3, Table A4, Table A5, Table A6, Table A7, Table A8, Table A9, and Table A10). Routine inpatient SSI surveillance, ICD-9-CM diagnosis code (Table A1, Table A2, Table A3, Table A4, Table A5, Table A6, Table A7, Table A8, Table A9, and Table A10) and antimicrobial drug exposure screening, and medical record review were performed as described. We used an antimicrobial interval of >6 days for breast procedures.

#### Phase 2–All Epicenters

One hospital from each of five Epicenters participated. Three of the five hospitals did not perform routine prospective inpatient surveillance for SSI after breast procedures. Methods for screening, sampling, estimating SSI rates, sensitivities, and positive predictive value were as described for cesarean deliveries.

## Results

A total of 8,739 CABG procedures, 7,399 caesarean deliveries, and 6,175 breast procedures were assessed (Table 1). In addition to routine prospective surveillance as described, 189–451 charts per procedure type were reviewed at each hospital. Hospital-specific results are shown in Table A1, Table A2, Table A3, Table A4, Table A5, Table A6, Table A7, Table A8, Table A9, and Table A10.

**Table 1:** Number of procedures and hospitals included in phase 1 (Eastern Massachusetts Epicenter) and phase 2 (all epicenters)

Procedures and phase	No. of procedures	No. of hospitals	Procedures per hospital (range)
Coronary artery bypass graft			
Phase 1	2,267	4	173–775
Phase 2	6,472	6	217–2,221
Cesarean delivery			
Phase 1	2,659	5	118–1,248
Phase 2	4,740	4	628–2,437
Breast procedures			
Phase 1	1,477	7	52–503
Phase 2	4,698	5	329–1,822

### CABG Procedures

#### Phase 1

The overall SSI rate based on confirmed infections detected by any of the three methods was 6.3% (Table 2). The sensitivities of routine prospective surveillance (79%) and antimicrobial drug exposure screening (80%) were essentially equal and both substantially exceeded that of ICD-9-CM diagnoses codes (61%) (Table 3). The positive predictive value of antimicrobial drug exposure (33%) was considerably lower than that for surveillance based on diagnosis codes (86%), which reflected the fact that 15% of patients met the antimicrobial drug exposure criteria compared with 4.5% of those who were assigned the screening diagnosis codes. The patients who met either the antimicrobial drug exposure or diagnosis code–screening criteria overlapped substantially, which resulted in the joint measure's having performance characteristics similar to that of antimicrobial drug exposure screening alone.

**Table 2:** SSI rates after CABG procedures, cesarean delivery, and breast procedures^a,b^

Procedure/phase	SSI rates detected by	
	% routine surveillance (SSI/ procedures) [95% CI]	Any method %^c^ (SSI/procedures) [95% CI]
CABG		
Phase 1	4.9 (112/2,267) [4.1%–5.9%]	6.3 (142/2,267) [5.3%–7.3%]
Phase 2	4.6 (298/6,472) [4.1%–5.1%]	7.7 (501/6,472) [7.1%–8.9%]
Combined	4.7 (410/8,739)	7.4 (643/8,739)
Cesarean delivery		
Phase 1	1.6 (43/2,659) [1.2%–2.2%]	4.1 (110/2,659) [3.4%–5.0%]
Phase 2	1.6 (49/3,065)^d^ [1.2%–2.1%]	5.5 (263/4,740) [4.8%–6.3%]
Combined	1.6 (92/5,724)	5.0 (373/7,399)
Breast procedures		
Phase 1	0.7 (10/1,477) [0.3%–1.2%]	0.9 (14/1,477) [0.5%–1.6%]
Phase 2	0.4 (7/1,765)^d^ [0.2%–0.8%]	2.3 (110/4,698) [2.1%–2.8%]
Combined	0.5 (17/3,242)	2.0 (124/6,175)

^a^Based on routine surveillance and routine surveillance plus screening for antimicrobial drug exposure, discharge diagnosis codes, or both. ^b^SSI, surgical site infection; CABG, coronary artery bypass graft; CI, confidence interval. ^c^Routine, antimicrobial exposure, diagnosis codes. ^d^The total number of procedures is noted for the hospitals that had performed routine surveillance.

**Table 3:** Sensitivity and positive predictive value of routine surveillance and screening by antimicrobial drug exposure, diagnosis codes, or both for identifying SSI after CABG procedures, cesarean delivery, and breast procedures^a^

Procedure/phase	Sensitivity^b^ (%)				Positive predictive value^b^ (%)		
	Routine surveillance	Antimicrobial exposure	Diagnosis code	Antimicrobial exposure and/or diagnosis code	Antimicrobial exposure	Diagnosis code	Antimicrobial exposure and/or diagnosis code
CABG							
Phase 1	79	80	61	87	33	86	35
Phase 2	59	91	54	93	36	84	36
Cesarean delivery							
Phase 1	39	90	48	96	42	61	42
Phase 2	38	84	78	97	37	67	38
Breast procedures							
Phase 1	71	71	50	79	19	58	20
Phase 2	33	94	70	96	33	79	33

^a^SSI, surgical site infections; CABG, coronary artery bypass graft. ^b^Compared to standard criteria comprised of all infections identified during prospective surveillance or medical record review.

The SSI classification assigned by hospital-based and external reviewers agreed for 107 (82%) of 130 procedures, with an overall κ coefficient of 0.66 (95% CI 0.53, 0.79). κ coefficients did not vary significantly across hospitals (p = 0.11).

#### Phase 2

The overall rate of confirmed SSI based on the combination of methods was 7.7% (Table 2); approximately one third of these were deep sternal SSI (SSI rates of 2.2% for deep sternal, 2.5% for superficial sternal, 3.1% for superficial donor site, and 0.2% for deep donor site SSI). In contrast to phase 1, antimicrobial drug exposure screening (>9 days) identified substantially more infections (sensitivity 91%) than routine surveillance (59%), which performed slightly better than coded discharge diagnoses (54%) (Table 3). The sensitivity did not vary meaningfully for different SSI types. The combination of a >9-day antimicrobial interval and discharge diagnoses identified 93% of SSI. The overall positive predictive values of antimicrobial drug screening, diagnosis code screening, and the combination of both were similar to those observed in phase 1, with higher values for diagnosis codes. The proportions of patients meeting the three screening criteria were also similar to the values observed in phase 1 (Table 4). A more liberal antimicrobial interval of >7 days negligibly increased sensitivity from 91% to 93%, reduced the positive predictive value to 30%, and increased the proportion of patients who met the criteria to 23% (Table A4). We observed no significant heterogeneity in the performance of screening by antimicrobial threshold to detect SSI among the six hospitals (p = 0.9).

**Table 4:** Percentage of patients who met the antimicrobial drug, diagnosis code–screening criteria, or both, after CABG procedures, cesarean delivery, and breast procedures^a^

Procedure and phase	% of patients meeting		
	Antimicrobial exposure criteria	Diagnosis code criteria	Antimicrobial exposure or diagnosis code criteria
CABG			
Phase 1	15.2	4.5	15.8
Phase 2	19.1	4.6	19.6
Cesarean delivery			
Phase 1	8.8	3.2	9.5
Phase 2	12.7	6.4	14.1
Breast procedures			
Phase 1	3.5	0.8	3.7
Phase 2	6.7	2.0	6.8

^a^CABG, coronary artery bypass graft.

### Cesarean Delivery Surveillance

#### Phase 1

The overall rate of confirmed SSI was 4.1%, based on the combination of methods (Table 2). An antimicrobial interval of >2 days identified 90% of infections, compared with diagnosis codes alone (48%) and routine surveillance (39%) (Table 3). Approximately 9% of patients met the antimicrobial drug exposure criterion, and 3.2% had one of the discharge diagnoses of interest (Table 3). The combination of antimicrobial drug exposure and diagnosis codes increased sensitivity slightly (96%) and was similar to antimicrobial drug exposure alone in predictive value and percentage of patients who met the criterion.

#### Phase 2

The overall infection rate was 5.5% (Table 2). The performance of the surveillance measures was similar to their performance in phase 1, in that an antimicrobial interval of >2 days identified substantially more infections (84%) than routine surveillance (38%, for the two hospitals that performed routine surveillance). Results of routine surveillance were comparable to those of antimicrobial drug exposure for deep incisional SSI (SSI rate of 0.3% for each), with superficial incisional SSI (0.3% vs. 1.3%) and endometritis (1.0% vs 2.6%), which accounted for the lower sensitivity of routine surveillance. In this phase, diagnosis codes (84% sensitivity) performed substantially better than routine surveillance and nearly as well as antimicrobial exposure. The positive predictive value to detect SSI was highest for diagnosis code–based screening (67%), and it was 37% for screening by antimicrobial drug exposure. The combination of antimicrobial exposure, diagnosis code–screening, or both improved sensitivity to 97% and had a positive predictive value similar to that of antimicrobial drug exposure alone.

As observed for CABG procedures, the proportions of patients who met screening criteria were similar for antimicrobial drug exposure alone (12.7%) and the combination of antimicrobial exposure and diagnosis codes (14.1%). A smaller proportion (6.4%) met only diagnosis code–screening criteria.

### Breast Surgery Surveillance

#### Phase I

The overall confirmed SSI rate was 0.9%, based on the combination of all three methods (Table 2). Routine surveillance and antimicrobial drug exposure screening identified 71% of SSI (Table 3), compared with 50% for diagnosis codes. The combination of antimicrobial drug exposure and diagnosis codes identified 79%. The positive predictive value to detect SSI was highest for diagnosis code–based screening (58%), and was 19% and 20%, respectively, for screening by antimicrobial exposure and screening by a combination of antimicrobial drug exposure and diagnosis codes. The percentages of patients who met antimicrobial exposure criteria, diagnosis code criteria, and a combination are listed in Table 4.

#### Phase 2

The overall SSI rate, based on all three methods, was higher (2.2%) in this phase. Antimicrobial drug exposure was the most sensitive measure; it identified 94% of infections, compared with 70% for diagnosis codes and 33% for routine surveillance (two hospitals' data). The sensitivity of routine surveillance was similar for both deep and superficial infections.

The positive predictive value for detecting SSI was highest for diagnosis codes (79%); the positive predictive value was 33% for antimicrobial exposure alone and for the combination of antimicrobial drug exposure and diagnosis codes. The proportions of patients who met screening criteria were similar with antimicrobial drug exposure alone and the combination of antimicrobial exposure and diagnosis codes (6.7% and 6.8%, respectively), with a smaller proportion (2.0%) who met only diagnosis code–screening criteria.

## Discussion

Many hospitals do not perform active SSI surveillance because it requires substantial resources. When hospitals do perform surveillance, our experience indicates that they often miss a substantial portion of infections. In the current study, many infections were missed after cesarean delivery and breast surgery, possibly because brief postoperative hospitalizations and infrequent readmissions for infection limited the efficiency of routine surveillance. The typical absence of microbiologic culture data associated with postcesarean endometritis may have also compromised the sensitivity of routine SSI surveillance after cesarean delivery. Furthermore, hospitals often cannot meaningfully compare their results with those from other hospitals because surveillance methods are not standardized. In contrast, surveillance based on antimicrobial drug exposure and diagnosis codes is objective and uses information that is collected routinely and is often available electronically. These factors may facilitate surveillance that performs uniformly over time and between institutions. We believe this method is likely to be widely applicable because the hospitals we studied had well-developed, independent surveillance programs and used a variety of different information technologies, yet all substantially improved their detection of SSI.

Overall, inpatient antimicrobial drug exposure was the best single measure for identifying SSI. We improved this measure's specificity by ignoring antimicrobial drugs administered on the operative and first postoperative days, when many patients receive perioperative antibiotic prophylaxis, and by omitting the large number of patients who received brief courses of antimicrobial drugs for other reasons. Even so, most patients identified by this method did not have conditions that met the CDC's SSI definitions. We previously observed that many of these patients without SSI are either "near misses," that is, they had signs and symptoms that prompted physicians to treat them as if they had an infection, or they had healthcare-associated infections at other sites (*2*). Therefore, above-threshold antimicrobial drug exposure can be a useful marker for clinically important postoperative illness that does not meet formal criteria for postoperative infection or for which documenting the medical record is insufficient to confirm a diagnosis.

In institutions such as the teaching and community hospitals in this study, infection control professionals will need to review from 4% to 20% of medical records to determine the SSI status for each patient who meets antimicrobial drug exposure or diagnosis code criteria, a percentage that is substantially lower than that required by routine surveillance. Moreover, confirming the status of each patient may not be necessary when the fraction of patients who meet the antimicrobial drug exposure criterion is within a stable range. Infection control professionals could reserve such assessments for instances when the elevation of this fraction above a specified threshold suggests the need for additional investigation.

The addition of diagnosis codes to antimicrobial drug–based surveillance improved sensitivity by 2% to 10%. Diagnosis codes improved screening for SSI after cesarean deliveries most markedly. In general, added sensitivity is likely not worth the extra effort currently required in most institutions to work with two different data sources. Screening for more codes might increase the sensitivity of this measure. The incremental value added by other types of information, such as microbiologic data, which have been studied by others (*5**,**6*), is unknown. Including such information, however, would add complexity to the process of acquiring and evaluating data. When additional automated data sources become widely available, the contribution they can make should be determined.

This study has several limitations. First, we may have missed some infections because we did not review the medical records from all patients or because the medical records had insufficient documentation. Second, before this surveillance method can be extended beyond these three surgical procedures, specific antimicrobial intervals will need to be evaluated for other procedures. Thus, additional studies are needed to assess the usefulness of this approach for other surgical procedures. Third, and perhaps most importantly, our studies did not address postdischarge surveillance for SSI, except when patients were readmitted to the same hospital. Therefore, assessment of inpatient antimicrobial drug exposure is only useful as a substitute or enhancement for traditional methods for detecting SSI among inpatients. The important problem of detecting postdischarge infections in patients who are not readmitted to the same hospital must be addressed through other means (e.g., through the use of automated claims data) (*7*).

To reduce the number of SSI, we need to better understand their occurrence in all hospitals that perform operations, which is not possible by using current surveillance methods. One way to perform standardized surveillance in all hospitals would be to use relatively simple, broad-based surveillance among inpatients by monitoring antimicrobial drug exposure, together with more intensive surveillance of hospitals that appear to have high infection rates not explained by the difference in underlying patient risks for infection. Confirming a high case-mix adjusted infection rate would prompt evaluation of opportunities to improve policies, procedures, and training for personnel. Our studies indicate that monitoring inpatient antimicrobial drug exposure, possibly in combination with diagnosis codes for certain procedures, identifies more infections, requires fewer resources, and may be more easily standardized than conventional surveillance. These methods might replace conventional surveillance in some situations or, at a minimum, be used to focus valuable surveillance resources on patients most likely to have SSI.

## Appendix

**Table A1:** ICD-9-CM procedure and diagnosis codes to identify study procedures and screen diagnosis codes

ICD-9-CM codes	Description
Procedure codes	
CABG	
36.10–36.14	Aortocoronary bypass of coronary artery/arteries
36.15–36.17	Internal mammary-coronary artery bypass
36.19	Other bypass anastomosis for heart revascularization
36.2	Heart revascularization by arterial implant
Cesarean delivery	
74.0–74.2, 74.4	Cesarean section (classical, low cervical, extraperitoneal, of other specified type)
74.9, 74.91, 74.99	Cesarean section of unspecified type
Breast procedures	
85.23	Subtotal mastectomy
85.31–85.32	Reduction mammoplasty
85.33–85.36	Subcutaneous mammectomy
5.4, 85.41–85.48	Mastectomy (unilateral or bilateral, simple or radical)
5.50	Augmentation mammoplasty
5.53	Unilateral breast implant
5.54	Bilateral breast implant
5.6	Mastopexy
5.7	Total reconstruction of breast
Diagnosis codes	Description
998.5, 998.51, 998.59	Postoperative infection
674.32, 674.34	Other complications of obstetrical surgical wounds
670.02, 670.04	Major puerperal infection

^a^ICD-9-CM, International Classification of Diseases 9th Revision Clinical Modification; CABG, coronary artery bypass graft procedures.

**Table A2:** SSI rates^a^ after CABG procedures^b,c^

Hospital	SSI rates based on (%)	
	Routine surveillance	Any method (routine, antimicrobial exposure, discharge diagnoses)
1A	2.2	2.6
1B	2.3	3.5
1C	5.9	7.4
1D	7.7	9.9
Phase 1 pooled (SSI/procedures)	4.9 (112/2,267)	6.3 (142/2,267)
2A	2.4	6.1
2B	2.5	6.6
2C	3.5	6.0
2D	5.3	8.2
2E	6.1	9.6
2F	6.5	8.8
Phase 2 pooled (SSI/procedures)	4.6 (298/6,472)	7.7 (501/6,472)

^a^The numbers of SSI (surgical site infection) and procedures are not specified by hospital to maintain confidentiality of the hospitals' identities. ^b^CABG, coronary artery bypass graft. ^c^On the basis of routine surveillance and routine surveillance plus antimicrobial exposure or diagnosis code screening.

**Table A3:** Sensitivity and positive predictive value of routine surveillance, and antimicrobial exposure screening using a 9-day antimicrobial interval or diagnosis code screening for identifying SSI after CABG procedures^a^

Hospital	Sensitivity				Positive predictive value		
	Routine surveillance	Antimicrobial exposure	Diagnosis codes	Antimicrobial exposure or diagnosis codes	Antimicrobial exposure	Diagnosis codes	Antimicrobial exposure or diagnosis codes
1A	85	75	45	75	33	75	14
1B	67	83	67	100	56	89	55
1C	80	82	57	84	47	86	47
1D	78	81	83	92	37	83	40
Pooled	0.79	0.80	0.61	0.87	0.33	0.86	0.35
2A	42	97	67	97	30	100	30
2B	41	100	78	100	35	78	33
2C	58	100	55	100	39	87	39
2D	65	92	57	95	33	89	33
2E	63	78	28	83	43	73	44
2F	74	100	53	100	39	77	39
Pooled	59	91	54	93	36	84	36

^a^SSI, surgical site infections; CABG, coronary artery bypass graft.

**Table A4:** Percentage of patients who underwent CABG procedures and who met the antimicrobial exposure or diagnosis code screening criteria^a^

Hospital	% of CABG patients with			
	9-day antimicrobial threshold	7-day antimicrobial threshold	Diagnosis code criteria	9-day antimicrobial or diagnosis code criteria (%)
1A	13.7	–^b^	2.2	13.8
1B	21.0	–^b^	7.4	22.2
1C	12.9	–^b^	4.9	13.2
1D	5.2	–^b^	3.5	6.4
Pooled	15.2	–^b^	4.5	15.8
2A	18.6	18.9	3.7	18.6
2B	17.3	21.0	6.2	18.2
2C	15.0	17.0	3.9	15.0
2D	22.9	31.5	5.3	23.6
2E	17.4	18.2	3.7	17.9
2F	22.6	25.3	6.0	22.6
Pooled	19.1	23.1	4.6	19.6

^a^CABG, coronary artery bypass graft. ^b^7-day antimicrobial threshold was not evaluated.

**Table A5:** SSI rates^a^ after cesarean delivery^b,c^

Hospital	SSI rates (%)	
	Routine surveillance	Any method (routine, antimicrobial exposure, diagnosis codes)
1A	2.2	3.8
1B	1.5	5.0
1C	0.9	2.7
1D	0.8	1.7
1E	5.3	6.4
Phase 1 pooled (SSI/procedures)	1.6 (43/2,659)	4.1 (110/2,659)
2A	0.3	3.7
2B	1.9	4.3
2C	NA	5.9
2D	NA	8.2
Phase 2 pooled (SSI/procedures)	1.6 (49/3,065)	5.5 (263/4,740)

^a^The numbers of SSI and procedures by hospital are not specified to maintain confidentiality of the hospitals' identities. ^b^SSI, surgical site infection; NA, routine SSI surveillance for this procedure type was not performed at this hospital. ^c^On the basis of routine surveillance versus routine surveillance plus screening by antimicrobial exposure or diagnosis code criteria.

**Table A6:** Sensitivity and positive predictive value of routine surveillance and antimicrobial exposure screening^a^

Hospital	Sensitivity (%)				Positive predictive value (%)		
	Routine surveillance	Antimicrobial exposure	Diagnosis codes	Antimicrobial exposure or diagnosis code	Antimicrobial exposure	Diagnosis codes	Antimicrobial exposure or diagnosis code
1A	58	83	50	92	40	50	38
1B	30	94	48	98	52	73	52
1C	32	86	54	95	27	43	27
1D	50	100	0	100	20	0	20
1E	82	82	45	91	56	83	59
Phase 1 pooled	39	90	48	96	42	61	42
2A	9	87	100	100	45	49	39
2B	44	73	75	100	48	100	56
2C	NA	98	80	100	21	40	20
2D	NA	89	72	92	46	81	44
Phase 2 pooled	38	84	78	97	37	67	38

^a^SSI, surgical site infections; NA, routine SSI surveillance for this procedure type was not performed at this hospital. ^b^Using a 2-day antimicrobial interval or diagnosis code screening for identifying SSI after cesarean delivery.

**Table A7:** Percentage of patients who underwent cesarean delivery and who met the antimicrobial exposure or diagnosis code screening criteria

Hospital	% of cesarean deliveries with		
	2-day antimicrobial exposure criterion	Diagnosis code criteria	2-day antimicrobial exposure or diagnosis code criteria
1A	7.9	3.8	9.2
1B	9.1	3.3	9.4
1C	8.8	3.5	9.7
1D	8.5	0	8.5
1E	9.4	3.5	9.9
Pooled	8.8	3.2	9.5
2A	7.0	7.5	9.4
2B	6.5	3.3	7.7
2C	27.4	11.8	28.6
2D	20.0	9.2	21.2
Pooled	12.7	6.4	14.1

**Table A8:** SSI rates^a^ after breast procedures^b,c^

Hospital	SSI rates (%)	
	Routine surveillance	Any method (routine, antimicrobial, discharge diagnoses)
1A	0	0
1B	1.0	1.2
1C	1.2	2.1
1D	0	0
1E	0	0
1F	1.3	1.3
1G	0	0
Phase 1 pooled (SSI/procedures)	0.7 (10/1,477)	0.9 (14/1,477)
2A	0.3	1.6
2B	0.4	0.9
2C	NA	3.6
2D	NA	3.3
2E	NA	1.8
Phase 2 pooled (SSI/procedures)	0.4 (7/1,765)	2.3 (110/4,698)

^a^The numbers of SSI and procedures by hospital are not specified to maintain confidentiality of the hospitals' identities. ^b^SSI, surgical site infection; NA, routine SSI surveillance for this procedure type was not performed at this hospital. ^c^On the basis of routine surveillance versus routine surveillance plus screening by antimicrobial exposure or diagnosis code criteria.

**Table A9:** Sensitivity and positive predictive value of routine surveillance and antimicrobial exposure screening^a,b^

Hospital	Sensitivity (%)				Positive predictive value (%)		
	Routine surveillance	Antimicrobial exposure	Diagnosis codes	Antimicrobial exposure and/or diagnosis codes	Antimicrobial exposure	Diagnosis codes	Antimicrobial exposure and/or diagnosis codes
1A	–	–	–	–	0	0	0
1B	83	50	50	50	60	100	60
1C	57	86	43	100	40	75	44
1D	–	–	–	–	0	–	0
1E	–	–	–	–	0	–	0
1F	100	100	100	100	33	100	33
1G	–	–	–	–	0	–	0
Pooled	71	71	50	79	19	58	20
2A	0	100	50	100	20	75	20
2B	50	100	42	100	33	45	33
2C	NA	92	75	92	38	56	34
2D	NA	95	82	100	37	96	38
2E	NA	80	40	100	29	62	27
Pooled	33	94	70	96	33	79	33

^a^SSI, surgical site infection; NA, not applicable, –, no SSI identified at this hospital; NA, routine SSI surveillance for this procedure type was not performed at this hospital. ^b^Using a 6-day antimicrobial interval or diagnosis code screening for identifying SSI after breast procedures.

**Table A10:** Percentage of patients who underwent breast surgery and who met the antimicrobial or diagnosis code screening criteria

Hospital	% of breast surgery with		
	6-day antimicrobial exposure criterion	Diagnosis code criteria	6-day antimicrobial exposure or diagnosis code criteria
1A	6.2	1.4	6.5
1B	1.0	0.6	1.0
1C	4.4	1.2	4.7
1D	1.5	0	1.5
1E	0	0	0
1F	3.8	1.3	3.8
1G	10.2	0	10.2
Pooled	3.5	0.8	3.7
2A	7.9	1.0	7.9
2B	2.6	0.8	2.6
2C	8.8	4.9	9.7
2D	8.5	2.8	8.7
2E	6.3	1.7	6.5
Pooled	6.7	2.0	6.8
